# Clinical Lecture upon Plaster of Paris Jacket

**Published:** 1878-02

**Authors:** Julius F. Miner


ART. II.—*Clinical Lecture upon Plaster of Paris Jacket* in Buffalo General Hospital. By J. F. MINER, M. D.

GENTLEMEN:—Those of you who have not seen the so-called Plaster Paris Jacket will be interested to see it applied. Dr. W. W. Miner and Mr. Stockton my colleagues in the Hospital are experts in the application, and I will stand back and invite your attention to them.

This method of treating spinal curve has recently been revived and has taken the surgical world by storm. It has been written, spoken and sung, until nearly all have got, or have tried to get a benefit out of it. The profession have applied it as something new. Some members of it go so far as to announce it as a specialty and "don't do any thing else," I learn that even some of the clergy in our own city have announced its results as miraculous, and hardly to be believed. It has grown in favor so rapidly that considerate men begin to expect its decadence and final demise, to sleep another three hundred years coming up again hereafter to astonish the world as something new. You observe how it is made and how it is applied. This case is well adapted to its application and I hope this jacket has some advantages over all other forms of dressing the spine, in posterior or angular curve. As the patient is suspended by the neck and shoulders you can see how the spine is forced into line by the weight of the body below the curve. Posterior or angular curve is almost always caused by caries or softening of the vertebral bodies, and you will see how straightening it will throw the weight of the body above the curve upon the transverse processes of the vertebral bones, which are not carious or softened and are better calculated to sustain this weight, relieving the softened bone tissue now unable to sustain itself under pressure. The philosophy of it is apparent to you all, and it has some advantages which are worth consideration. It is cheaper, more readily applied, available to all surgeons wherever located and so far as tested, seems quite efficient in the treatment of posterior spinal curve.

Prof. Sayer, of New York, has distinguished himself as the champion of Plaster of Paris Jackets, and quite a number of younger and much less experienced surgeons have copied his example and somewhat like him cried out lustily *Eureka!* There are however several points remaining unsettled, before we really know "we have found it." Dr. Sayer has called attention to it with most unexampled energy and ability, and is justly entitled to the thanks of the profession and public. He has accomplished great good by calling attention to the true principles which govern in these cases, formerly neglected, now, everywhere better understood and better treated.

You will ask, "will it cure angular curve of the spine?" No, it will not in many instances *cure* spinal curve, but it will generally relieve and benefit, some cases almost entirely cure. I have heretofore called your attention to the pathological conditions of this disease, by which you will see that perfect restoration is often times wholly impossible, and to claim it, absurd. Will this jacket effect as good or better results than other surgical appliances? Plaster of paris is not wholly unobjectionable as a dressing, but until we find something better we must use it. Starch or Dextrine bandage similarly applied has some advantages over plaster, is cleaner and more comfortable and may be made to answer nearly the same purpose. The indication is now well understood, relief from *pressure* and *support*. The *best* means for accomplishing it is being discovered and demonstrated, possibly *is* discovered and fully demonstrated.

